# Vascular Remodelling Relates to an Elevated Oscillatory Shear Index and Relative Residence Time in Spontaneously Hypertensive Rats

**DOI:** 10.1038/s41598-017-01906-x

**Published:** 2017-05-17

**Authors:** Zhiyan Chen, Haiyi Yu, Yue Shi, Minjia Zhu, Yueshen Wang, Xi Hu, Youyi Zhang, Yu Chang, Ming Xu, Wei Gao

**Affiliations:** 1 Department of Cardiology, Peking University Third Hospital and Key Laboratory of Cardiovascular Molecular Biology and Regulatory Peptides, Ministry of Health, Key Laboratory of Molecular Cardiovascular Sciences, Ministry of Education and Beijing Key Laboratory of Cardiovascular Receptors Research, Beijing, 100191 China; 2 College of Life Science and Bioengineering, Beijing University of Technology, Beijing, 100124 China

## Abstract

Haemodynamic disorders are common clinical findings in hypertension and lead to adverse cardiovascular events. However, the haemodynamic conditions in hypertension models are poorly understood. This study aimed to observe the characteristics of haemodynamics in spontaneously hypertensive rats (SHRs) and antihypertensive-treated SHRs. Twenty-four adult male SHRs and Wistar-Kyoto rats (WKYs) were randomly divided into four groups and treated for 7 days as follows: WKY-CON (WKYs + saline), WKY-NIF (WKYs + nifedipine, 50 mg/kg/day), SHR-CON (SHRs + saline), and SHR-NIF (SHRs + nifedipine). Aortic computational fluid dynamics (CFD) models were simulated to obtain the haemodynamic parameters. We found that in the hypertensive (SHR-CON) and blood pressure-controlled (SHR-NIF) groups, the oscillatory shear index (OSI) and relative residence time (RRT), which are key haemodynamics indices, were markedly elevated. Furthermore, there was a correlation between both the elevated OSI and RRT with the vascular wall thickening in regions near the inner wall of the aortic arch. Our research demonstrates that haemodynamics remains disturbed even if the blood pressure is normalized. In addition, vascular remodelling may play an important role in maintaining elevated OSI and RRT values.

## Introduction

Haemodynamics caused by blood flow generates multiple mechanical forces that directly act on the endothelial surface of vessel walls. Normal haemodynamic conditions guide development during embryogenesis and remodelling to optimize blood flow to tissues throughout postnatal and adult life. However, disorder in haemodynamics leads to the exacerbation or acceleration of cardiovascular diseases^1^.

Haemodynamic disorder is a hallmark of hypertension. Blood pressure (BP) reduction is positively associated with the magnitude of risk reduction in recurrent cerebrovascular and cardiovascular events^2^. However, patients with controlled hypertension are still at high risk for developing comorbid cardiovascular diseases^3^. According to previous studies, vascular stiffness was significantly greater in controlled hypertensive patients than in healthy normotensive subjects^4, 5^. Furthermore, it was reported that the stiffness in the vessel wall increased the magnitude of turbulent fluctuating velocities^6^, suggesting the possible contribution of vascular wall remodelling to disrupted haemodynamics. The importance of vascular wall remodelling in haemodynamics has been shown; however, whether it contributes to haemodynamic disorders in BP-controlled hypertension models is poorly understood.

Computational fluid dynamics (CFD) is an appropriate mathematical method that mimics three-dimensional (3D) imaging of haemodynamics in vessels and is widely used in observing vascular diseases^7, 8^. The value and distribution of the wall shear stress (WSS), oscillatory shear index (OSI) and relative residence time (RRT) derived from 3D imaging analyses reveal the characteristics of haemodynamics in arteries^9, 10^. Low WSS was observed in bifurcations and associated with plaque formation in atherosclerosis^11^. Elevated OSI and RRT levels were found in post-stenotic region of vessels and were reported to be related to endothelial dysfunction^9^. In recent decades, CFD has been used to mimic the development of aneurysms and aortic dissection in aorta-related diseases^7, 8^; however, whether CFD could provide clues of regional haemodynamic conditions in hypertensive individuals is unknown.

Based on CFD technology, we designed *in vivo* experiments to identify the changes in the haemodynamics in hypertensive rats and in blood pressure-controlled hypertensive rats. Moreover, blood flow characteristics were visualized by timely CFD analyses. In the present study, crossing and integrating medical and mathematical models revealed a relationship between haemodynamic parameters (e.g., OSI and RRT) and vascular remodelling indicators (e.g., the thickness of vessel wall and elastin layer) under hypertensive conditions.

## Results

### Analysis of BP in hypertensive and antihypertensive-treated SHRs

Nifedipine, a potent L-type Ca^2+^ channel blocker, is a common antihypertensive drug and is highly selective for vascular smooth muscle cells^12^. We chose nifedipine as the treatment to control BP in our experiments. The BP in SHRs and WKYs after a 7-day treatment with either nifedipine or saline was detected. Compared with the WKY-CON group, the systolic blood pressure (SBP), diastolic blood pressure (DBP) and pulse pressure (PP) of the SHR-CON group were significantly higher (SBP: 217 ± 14 mmHg *vs* 125 ± 8 mmHg, *P* < 0.001; DBP: 164 ± 17 mmHg *vs* 90 ± 9 mmHg, *P* < 0.001; PP: 53 ± 7 mmHg *vs* 36 ± 4 mmHg, *P* < 0.01; Table 1). In addition, SBP, DBP and PP were ameliorated to normal levels in SHRs treated with nifedipine (SHR-CON *vs* SHR-NIF, SBP: 217 ± 14 mmHg *vs* 125 ± 10 mmHg, *P* < 0.001; DBP: 164 ± 17 mmHg *vs* 90 ± 16 mmHg, *P* < 0.001; PP: 53 ± 7 mmHg *vs* 34 ± 9 mmHg, *P* < 0.01; Table 1). This routinely used hemodynamic index, blood pressure, showed no difference between WKY-CON and SHR-NIF group, suggesting that the hypertensive rats had achieved the goal of antihypertensive treatment.

**Table 1:** Characteristics of the blood pressure in SHRs and WKYs.

	CON		NIF	
	WKY-CON	SHR-CON	WKY-NIF	SHR-NIF
W (g)	407 ± 15	368 ± 23**	403 ± 12	376 ± 17*^,†^
HR (beats/min)	271 ± 24	378 ± 23***	319 ± 47*	379 ± 35***^,††^
SBP (mmHg)	125 ± 8	217 ± 14***	111 ± 16	125 ± 10^###^
DBP (mmHg)	90 ± 9	164 ± 17***	73 ± 19	90 ± 16^###^
PP (mmHg)	36 ± 4	53 ± 7**	39 ± 12	34 ± 9^##^

Values are presented as the mean ± SD. (n = 6). *P < 0.05, **P < 0.01, ***P < 0.001, *vs* WKY-CON. ^##^ *P* < 0.01, ^###^ *P* < 0.001, *vs* SHR-CON. ^†^ *P* < 0.05, ^††^ *P* < 0.01, *vs* WKY-NIF. W, weight; HR, heart rates; SBP, systolic blood pressure; DBP, diastolic blood pressure; PP, pulse pressure; CON, control; NIF, nifedipine.

### Analysis of flow peak velocity in hypertensive and antihypertensive-treated SHRs

Compared to the WKY-CON group, the peak flow velocity in the ascending aorta (ASPV) detected with ultrasound imaging was significantly higher in the SHR-CON group (ASPV: 2047 ± 163 mm/s *vs* 1521 ± 92 mm/s, *P* < 0.001). In addition, the peak velocity in the aortic arch (AOPV) was lower in the SHR-CON group (SHR-CON *vs* WKY-CON, AOPV: 499 ± 78 mm/s *vs* 1038 ± 97 mm/s, *P* < 0.05, Table 2). Treatment with nifedipine significantly increased the ASPV in the SHR-NIF group compared with the values in the SHR-CON group (ASPV: 2239 ± 205 mm/s *vs* 2047 ± 163 mm/s, *P* < 0.05; Table 2).

**Table 2:** General features of the flow velocity in the aortas of experimental rats.

	CON		NIF	
	WKY-CON	SHR-CON	WKY-NIF	SHR-NIF
ASPV (mm/s)	1521 ± 92	2047 ± 163***	1846 ± 118**	2239 ± 205^#,^***^,†††^
AOPV (mm/s)	1038 ± 97	499 ± 78*	1324 ± 51	987 ± 109^†^

Values are presented as the mean ± SD. (n = 6). *P < 0.05, **P < 0.01, ***P < 0.001, *vs* WKY-CON. ^#^ *P* < 0.05, *vs* SHR-CON. ^†^ *P* < 0.05, ^†††^ *P* < 0.001, *vs* WKY-NIF. ASPV, ascending aorta peak velocity; AOPV, aortic arch peak velocity; CON, control; NIF, nifedipine.

### Alterations in TAWSS, OSI, RRT in aorta in hypertensive and antihypertensive-treated SHRs

To explore the precise haemodynamics in the aortic regions, CFD models were established, and the value and distribution of the WSS, OSI and RRT were derived from those models. Time averaged WSS (TAWSS) is defined as the average WSS values over one cardiac cycle. Previous studies have demonstrated that low TAWSS values represent haemodynamic disorder leading to plaque formation and vessel wall thickening. Compared with the WKY-CON group, the ratio of the area with low TAWSS (<20 Pa) in the aorta was significantly higher in the SHR-CON group (SHR-CON *vs* WKY-CON: 75.03 ± 6.96% *vs* 56.16 ± 12.56%, *P* < 0.01; Supplementary Fig. S1). Treatment with nifedipine obviously lowered the ratio of the area with low TAWSS (SHR-CON *vs* SHR-NIF, 75.03 ± 6.96% *vs* 59.25 ± 8.67%, *P* < 0.05; Supplementary Fig. S1).

**Figure 1:**
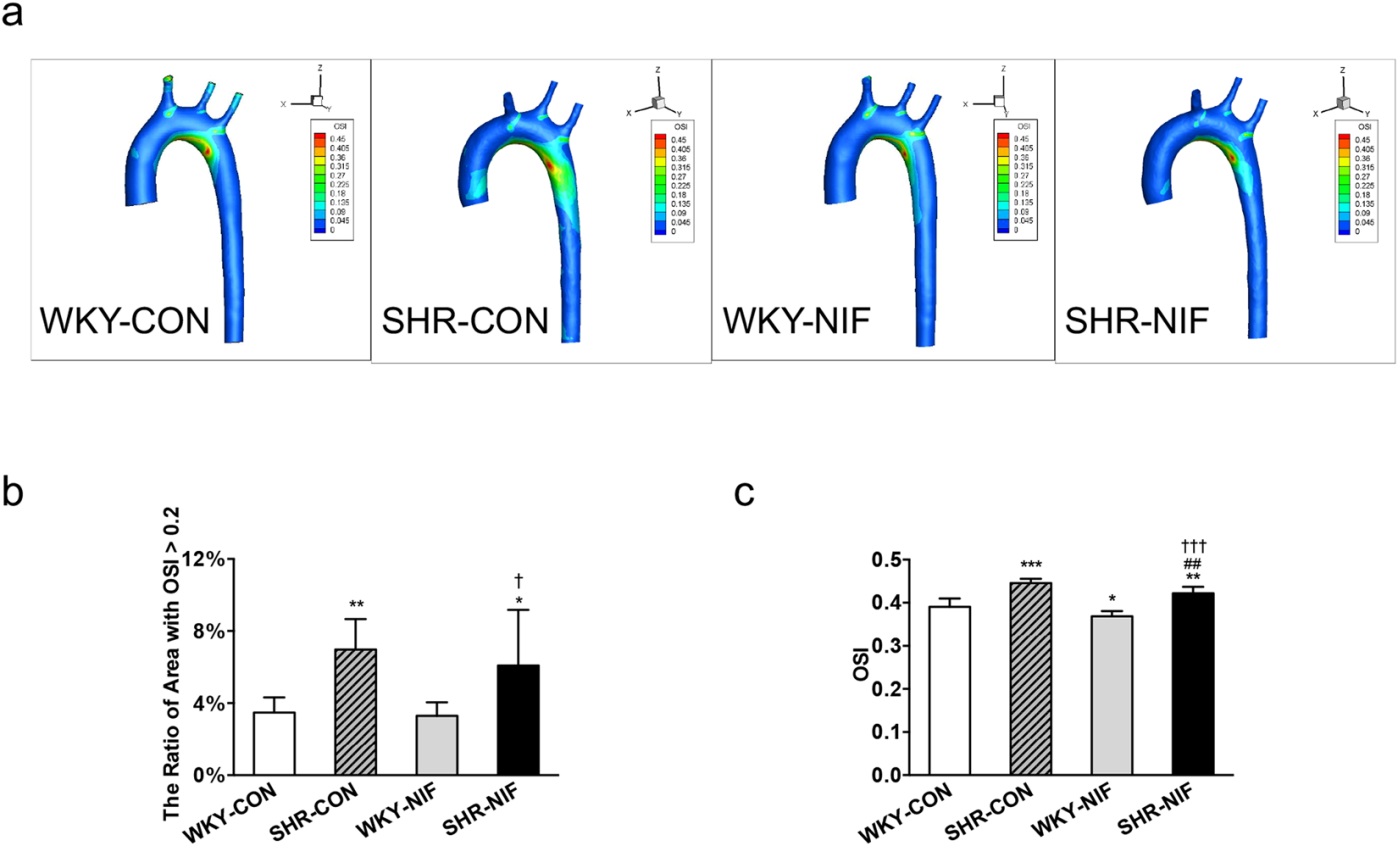
The OSI was significantly elevated in antihypertensive-treated SHRs compared with WKYs. (**a**) Contour plots of the OSI values averaged over one cardiac cycle analysed from aorta models in SHRs and WKYs. The OSI scale ranges from 0 (dark blue) to 0.5 (red). (**b**) The ratio of the area with an OSI > 0.2 of the whole geometry and (**c**) the OSI values in a region near the inner wall of the aortic arch are shown. Values are presented as the mean ± SD. (n = 6). *P < 0.05, **P < 0.01, ***P < 0.001, *vs* WKY-CON. ^##^ *P* < 0.01, *vs* SHR-CON. ^†^ *P* < 0.05, ^†††^ *P* < 0.001, *vs* WKY-NIF. CON, control; NIF, nifedipine.

The contours of OSI showed a large variation in the aorta models among groups (Fig. 1a). For all cases, the high values of OSI (red) was found near the inner vascular wall. High level of OSI represents detrimental flow conditions, and regions with an OSI > 0.2 are prone to vascular wall dysfunction^13^. In the present study, the ratio of an area with an OSI > 0.2 for the whole model was demonstrated to be significantly higher in the SHR-CON group compared to the WKY-CON group (SHR-CON *vs* WKY-CON: 6.98 ± 1.68% *vs* 3.47 ± 0.84%, *P* < 0.01, Fig. 1b). However, the antihypertensive treatment failed to attenuate the ratio of the area with an OSI > 0.2 (SHR-CON *vs* SHR-NIF, 6.98 ± 1.68% *vs* 6.09 ± 3.09%, Fig. 1b). Additionally, in the region near the inner wall of the aortic arch, the OSI values were markedly higher in the SHR-NIF group than in the WKY-CON group (SHR-NIF *vs* WKY-CON, 0.42 ± 0.02 *vs* 0.39 ± 0.02, *P* < 0.01, Fig. 1c).

**Figure 2:**
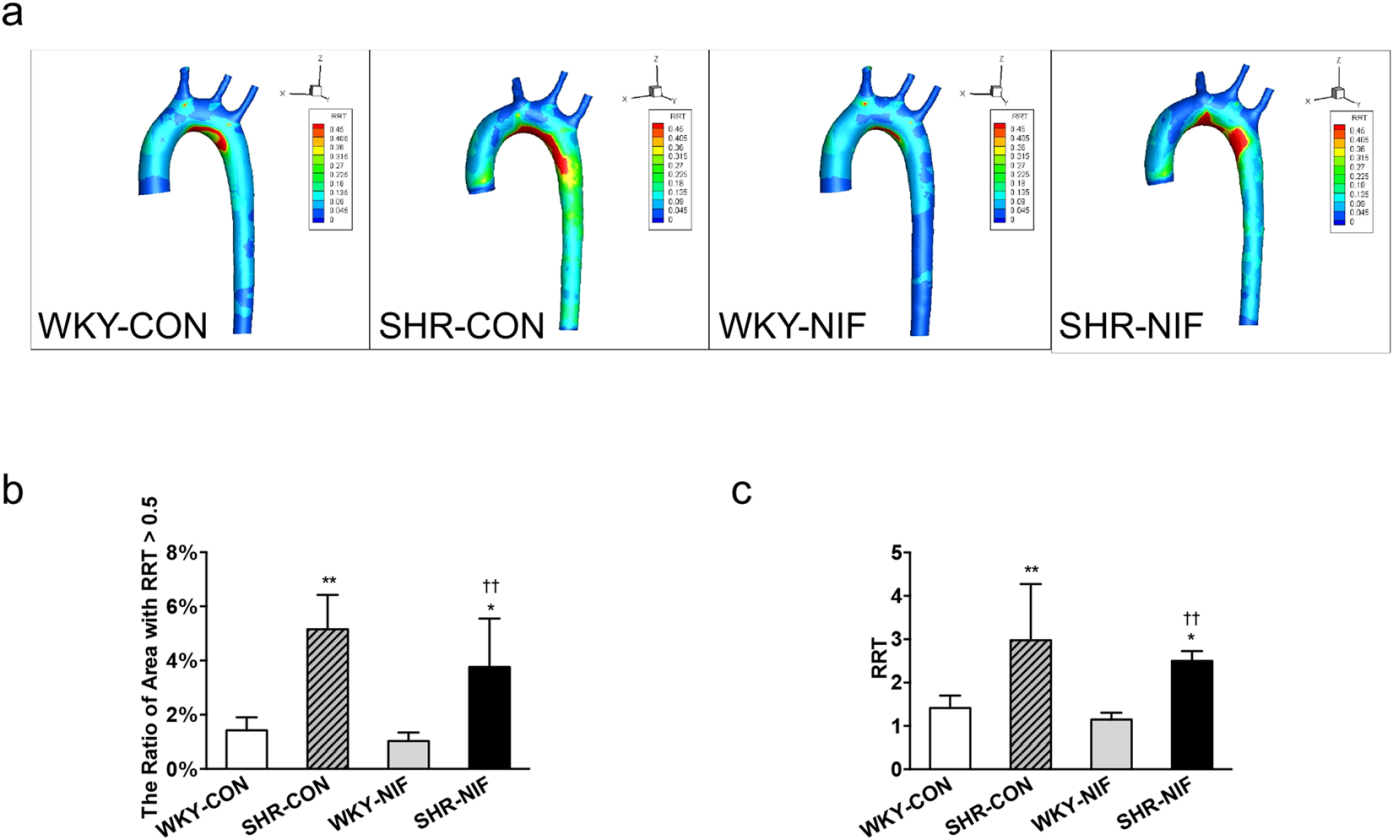
The RRT was obviously increased in antihypertensive-treated SHRs compared with WKYs. (**a**) Distribution of the RRT averaged over a cardiac cycle derived from aorta CFD models in SHRs and WKYs. The RRT scale ranges from 0 (dark blue) to 0.5 (red). (**b**) The ratio of the area with an RRT > 0.5 of the whole aorta model and (**c**) the RRT values in a region near the inner wall of the aortic arch were analysed. Values are presented as the mean ± SD. (n = 6). *P < 0.05, **P < 0.01, *vs* WKY-CON. ^††^ *P* < 0.01, *vs* WKY-NIF. CON, control; NIF, nifedipine.

There were large variations in the magnitudes of RRT distribution among SHRs and WKYs, and the distribution of RRT was in accordance with OSI, showed in Fig. 2a. The ratio of the areas with high RRT (>0.5) of the entire aorta is higher in SHR-CONs compared with WKY-CONs (5.16 ± 1.27% *vs* 1.43 ± 0.48%, *P* < 0.01, Fig. 2b), and no difference was shown between SHR-CONs and SHR-NIFs (SHR-CON *vs* SHR-NIF, 5.16 ± 1.27% *vs* 3.77 ± 1.79%, Fig. 2b). In the region near the inner wall of the aortic arch, the RRT values were significantly higher in the SHR-NIF group than in the WKY-CON group (SHR-NIF *vs* WKY-CON, 2.50 ± 0.23 *vs* 1.42 ± 0.29, *P* < 0.05, Fig. 2c).

### Assessment of the structure and function of the aorta after antihypertensive treatment

To determine the status of vascular stiffness in the aorta after antihypertensive treatment, regional pulse wave velocity (PWV) and vascular distension were measured by using ultrasound imaging. Compared to WKY-CONs, the aortic PWV was markedly higher, and distension (ΔD/D) of either the ascending aorta or aortic arch was obviously lower in SHRs and treated hypertensive rats (Supplementary Fig. S4).

**Figure 3:**
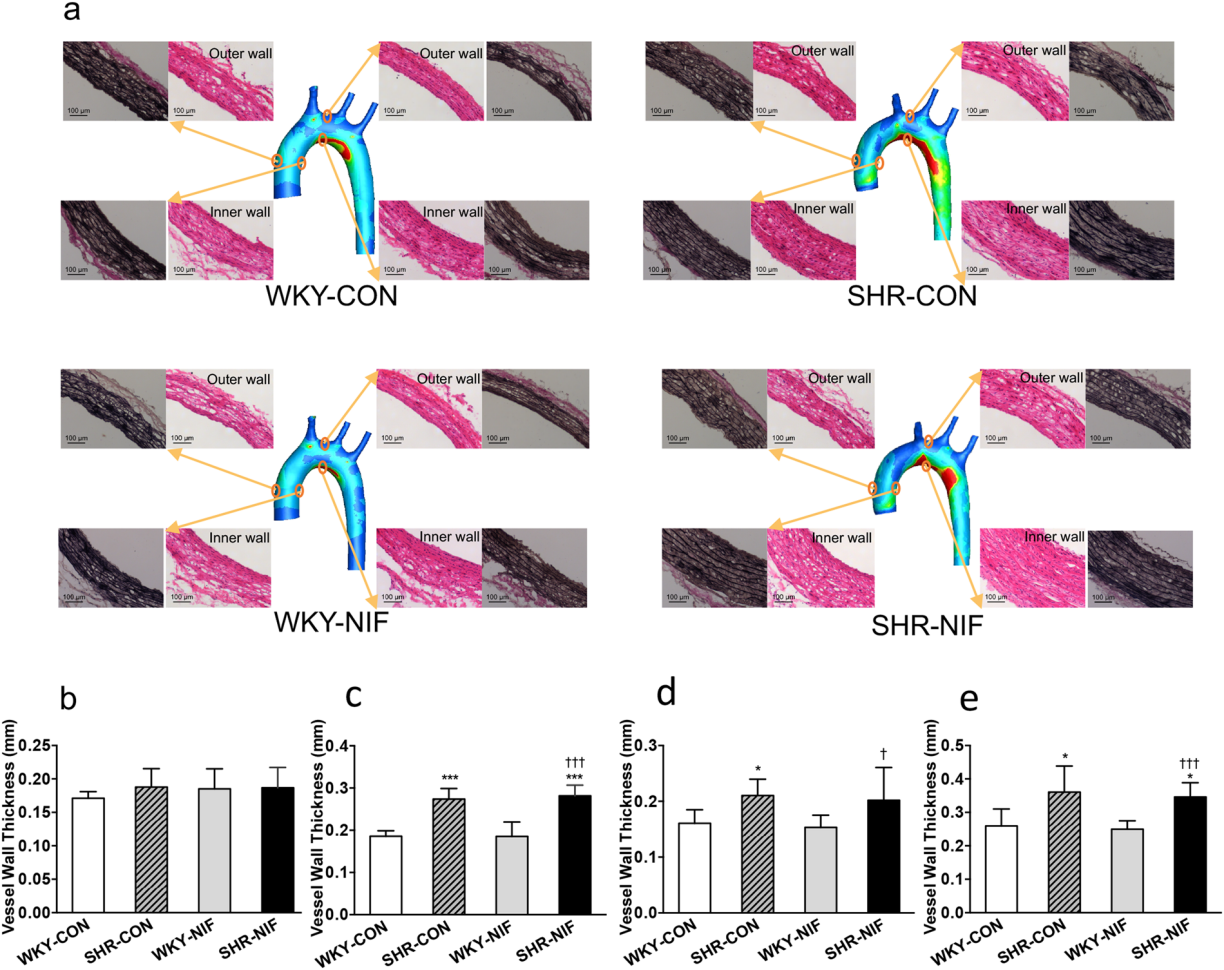
Arterial wall thickening and extensive elastin degradation were demonstrated near the inner wall of the aortic arch and was accompanied by elevated OSI and RRT values in hypertensive and antihypertensive-treated SHRs. (**a**) Histological analysis of cross-sections of the ascending aorta and aortic arch was performed with H&E and VVG staining in the WKY-CON, SHR-CON, WKY-NIF, and SHR-NIF groups. Quantitative analysis of the regional vessel wall thickness is shown in the (**b**) outer wall and (**c**) inner wall of the ascending aorta and the (**d**) outer wall and (**e**) inner wall of the aortic arch. Values are presented as the mean ± SD. (n = 6). *P < 0.05, ***P < 0.001, *vs* WKY-CON. ^†^ *P* < 0.05, ^†††^ *P* < 0.001, *vs* WKY-NIF. CON, control; NIF, nifedipine.

The aortic structure was determined by Haematoxylin and eosin (H&E) staining and Verhoeff-von Gieson (VVG) staining of the aortic tissue sections (Fig. 3a). Compared to WKY-CONs, the lumen diameter and cross-sectional area (CSA) of the aorta were larger in the SHR-CONs and SHR-NIFs (Supplementary Table S3). As an indicator of remodelling, the layer of extensive elastin degradation in the aorta was thicker in SHR-CONs and SHR-NIFs than in WKY-CONs (Supplementary Table S4 and Fig. 3a). Additionally, the aortic wall thickness (particularly near the inner wall of aortic arch) was significantly larger in the SHR-CON and SHR-NIF groups than in the WKY-CON (SHR-CON *vs* WKY-CON: 0.36 ± 0.08 mm *vs* 0.26 ± 0.05, *P* < 0.05; SHR-NIF *vs* WKY-CON: 0.35 ± 0.04 *vs* 0.26 ± 0.05, *P* < 0.05, Fig. 3e), suggesting that dysfunction and structural remodelling remained relatively unchanged after antihypertensive treatment.

### Correlations between haemodynamic parameters and vascular wall thickness in experimental rats

**Figure 4:**
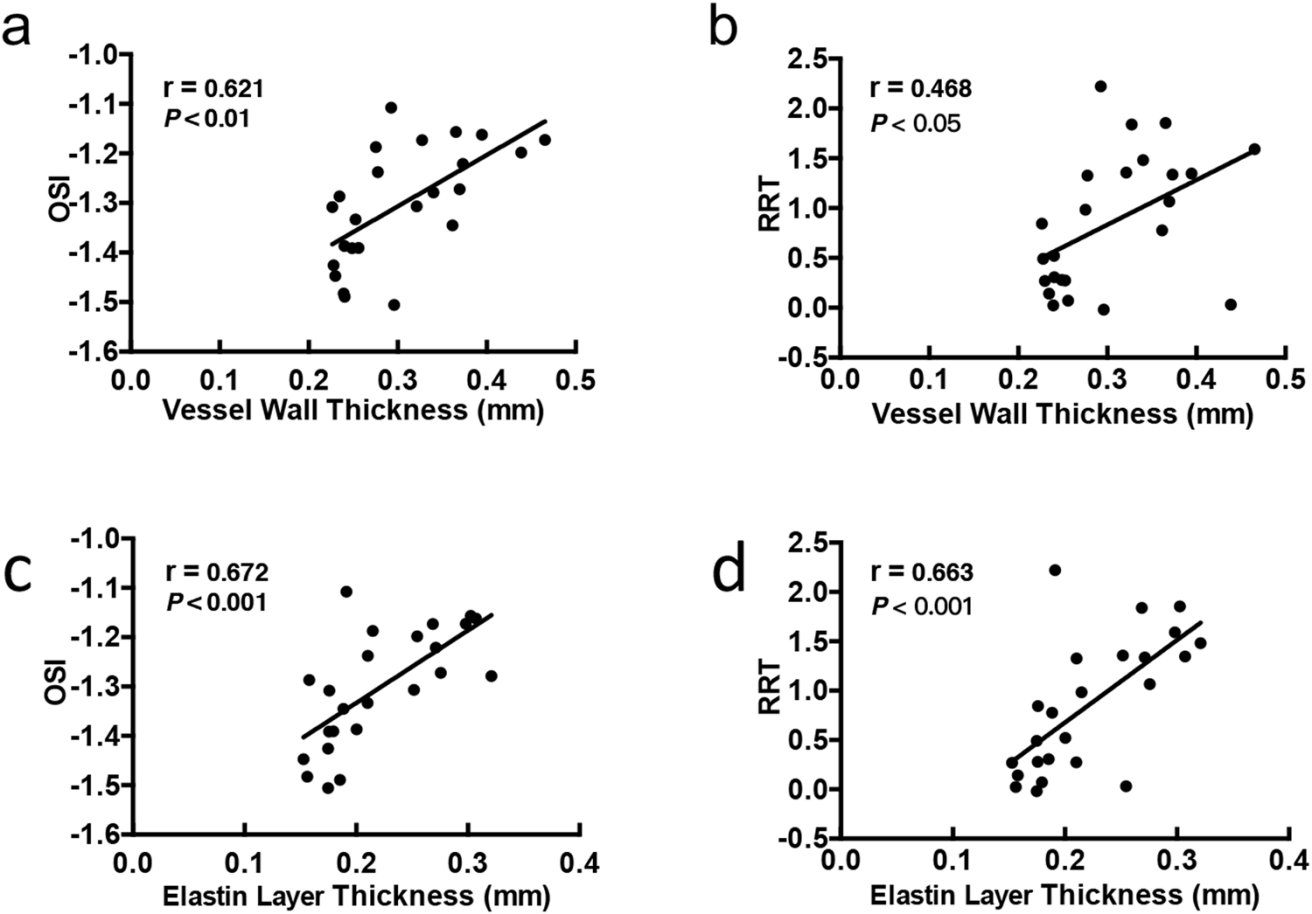
The correlations between haemodynamic parameters and vascular remodelling indicators in SHRs and WKYs. OSI and RRT were positively correlated to the vessel wall thickness (**a**,**b**) and to the elastin degradation layer thickness (**c**,**d**) near inner wall of aortic arch, respectively.

Based on the above results, we found that wall thickening and elastin degradation near the inner wall of the aortic arch were accompanied by elevated OSI and RRT levels in hypertensive and antihypertensive-treated SHRs (Figs 1c, 2c and 3a). Furthermore, the regression analysis showed that OSI and RRT were correlated to the vessel wall thickness and thickness of extensive elastin degradation layer near the inner wall of the aortic arch (OSI to vessel wall thickness: Pearson r = 0.621, *P* < 0.01; RRT to vessel wall thickness: Pearson r = 0.468, *P* < 0.05; OSI to elastin layer thickness: Pearson r = 0.672, *P* < 0.001; RRT to elastin layer thickness: Pearson r = 0.663, *P* < 0.001; Fig. 4).

## Discussion

In the present study, we found that after treatment with nifedipine, blood pressure was reduced to normal levels; however, haemodynamic disorder remains unchanged as indicated by the elevated OSI and RRT values. In addition, the regions with abnormal OSI and RRT were consistent with serious vascular remodelling, and the correlations were observed between elevated OSI and RRT values and the thickness of vessel wall and extensive elastin degradation, respectively. This finding is of clinical significance because vascular remodelling may be related to elevated OSI and RRT next to the vessel wall.

Ha﻿emodynamic disorder is a critical factor for increasing the risk of cardiovascular diseases and is a hallmark of hypertension. Low WSS values are often used as markers of disturbed flow to infer the pathophysiology of endothelial dysfunction^1, 14^. The WSS values in rodent aortas were roughly two orders of magnitude greater than in human (1–7 Pa)^1^, and there was no reference for the threshold of pathophysiologic values. In our study, the ratio of the area with low TAWSS (<20 Pa) in SHRs was increased by over 30% compared to that in WKYs, although the trend in regions selected in our study showed no differences. This result indicated a high risk of vascular injury in SHRs, which was consistent with previous studies^1, 15^. TAWSS < 20 Pa might be a pathophysiologic value for disturbed flow in SHRs. After reducing BP in the SHR-NIF group with nifedipine, the low values of TAWSS were obviously improved. This suggested that hypertension contributed to the lower WSS in the aorta which could be improved by antihypertensive treatment.

Elevated OSI levels are often accompanied by low TAWSS, and together they serve as indicators for vascular injury^9^. Blood flow induces the retrograde flow at the complex shape in the aortic arch when rotating through the aortic root to the descending aorta, causing a greater change in flow direction—particularly near the inner wall^16^. Along with oscillatory flow, there is also a prolonged residence time. These events are consistent with apparent disturbances in the OSI and RRT measured around the inner wall of aorta on OSI and RRT maps in our study. In addition, this region has a higher probability of developing lesions, as OSI values > 0.2 trend to the development of endothelial dysfunction^13^. Reallocated blood flow (probably induced by high BP) in SHRs led to approximately double the ratio of the area with elevated OSI (>0.2) compared with that in WKYs, which suggests high susceptibility of this area to wall lesion formation in hypertensive rats^11, 15^. Simultaneously, the area ratio of elevated RRT (defined as 0.5, the maximum of the colour scale) in SHRs was greater than 3-fold of that in WKYs. Therefore, we considered that there might be higher levels of OSI and RRT in the aorta of hypertensive rat. However, in BP-controlled SHRs, the drop in BP failed to attenuate the OSI and RRT values both for the whole aorta model that located around the inner curve of the aortic arch on maps and at regions near the inner wall of the aortic arch. This indicated that there might be some other underlying mechanisms that promote elevated OSI and RRT levels.

Blood flow interacts with the vessel wall as it passes through. Hypertension is one of the main diseases that causes vascular remodelling, which characterized by functional and structural changes in the vessels^17^. Serious stiffness and remodelling of the aortic wall were induced by an extended period of hypertension in SHRs, which was confirmed by the PWV, ΔD/D and histochemistry analyses^18, 19^ as well as by previous qualitative observations^15^. And nifedipine had little influence on aortic function and structure in the SHR-NIF group due to its strong effects on peripheral vessel. Vascular remodelling and stiffness provoke distinctive alterations in blood flow^6, 17^, which are in accordance with the findings regarding the difference in the blood flow velocity between SHR-NIFs and WKY-CONs in our study.

Upon significant arterial remodelling and stiffness in the aorta, oscillatory flow was demonstrated to be more severe in both hypertensive and antihypertensive-treated rats; this severity was characterized by the large area ratios and values of elevated OSI and RRT. BP might be largely responsible for the haemodynamic disturbance in the SHR-CON group. Vascular curvature might cause the quite difference in the values of OSI and RRT, which was considerably high in the aortic arch compared with the ascending aorta even in WKYs (Supplementary Figs S2 and S3). Vascular curvature provides spatial positions for vascular injury. However, hemodynamic disturbance was not improved in the aortic arch when we reduced the BP to the normal level in SHRs. It emphasized the importance of other underlying mechanisms, such as the serious vascular structural remodelling in this region. Arterial stiffness is independently associated with aortic flow reversal^20^. Aortic PWV alone is able to explain 13.2% of the total explainable variance of the aortic reverse flow^20^. A decrease in the fluid-solid coupled interaction might explain the severely disturbed flow in the SHR-NIF group, which was consistent with previous findings of significantly increasing turbulent kinetic energy in an ideal rigid ascending aorta model^6^. These findings indicated that the decreased interaction in artery regions might contribute to spatial susceptibility to lesions formation in hypertensive and antihypertensive individuals regardless of the drop in BP, especially in arteries with high tortuosity or bifurcations^21^. Reducing this increased risk required interventions, drugs or exercise, to directly improve vascular remodelling. Additionally, BP drop reduced the values of OSI and RRT in regions in the ascending aorta and the outer wall of the aortic arch in the SHR-NIF group; this was possibly due to the significantly higher flow velocity here or low baseline. Taken together, the OSI and RRT values were maintained at higher levels probably in the presence of serious vascular stiffness and remodelling, especially near the inner wall of aortic arch.

Wall thickness, extensive elastin degradation and lumen diameter enlargement were identified as good parameters to predict changes in the vascular structure, which confirms the results of previous studies^19, 22^. The relationships between vascular structure changes and haemodynamic parameters have been studied before. Cibis *et al*.^23^ believed that WSS derived from CFD models was inversely related with the WT of the carotid artery. However, Steinman *et al*.^24^ did not find a quantitative relationship between them. Maps of OSI and RRT were found to be consistent with plaque distributions in the aortic valve regurgitation (AR) mice^25^. Moreover, high OSI values were correlated with the development of intimal hyperplasia^26^. In the present study, the OSI and RRT values, but not TAWSS, qualitatively agreed with the thickness of vessel wall and extensive elastin degradation in regions near the inner wall of the aortic arch. It was believed that vascular remodelling might play a pivotal role in maintaining elevated OSI and RRT levels. Elevated OSI levels did not necessarily correlate with the relatively low levels of TAWSS as shown in Fig. 1, and Supplementary Fig. S1, which highlighted the importance of OSI as potential markers indicating serious vascular remodelling independent of low TAWSS levels^25^. Because the regions of arterial wall remodelling is identifiable via RRT sensitively coincided with the OSI in ours and previous study, elevated RRT also may be a useful metric for identifying vascular remodelling^27^. Thus, OSI and RRT are relatively sensitive haemodynamic indicators of regional vascular remodelling than TAWSS in our study.

Blood viscosity is another important factor in haemodynamics and may simultaneously increase the WSS and circulation resistance. It was reported that the different viscosity models estimated a less than 4% change on average for the different WSS metrics^28^. And with lack of accurate data for the blood viscosity in 8-month-old SHRs and WKYs, we set this metric at a commonly used value in CFD simulation of rats. Another limitation was that we did not test the relationship among elevated OSI, increased RRT and the changes of arterial wall thickness over time. In addition, we used CFD models which lost sight of the fluid-solid interaction in the process of simulation. CFD models may not as sensitive as fluid-structure interaction (FSI) models. This might lead to the different correlation coefficient between haemodynamic parameters and vascular wall thickness when compared to FSI as expected. An option to improve this might be provided by concerning the vascular structure factors using FSI models. Further study will be performed measuring the actual blood viscosity in animals, performing longitudinal observations and using FSI models.

In summary, haemodynamics remains disturbed even if the blood pressure is normalized. In addition, vascular remodelling may play an important role in maintaining elevated OSI and RRT levels.

## Methods

### Animals and treatments

All experiments were approved by the Ethical Committee for Animal Experiments of Peking University Health Science Centre in accordance with the National Institutes of Health Guide for the Care and Use of Laboratory Animals. Male SHRs and WKYs (Vital River Laboratory, Beijing, China) were randomly divided into four groups (n = 6 per group) and treated daily for 7 days as follows: WKY-CON (WKY + 0.9% saline), WKY-NIF (WKY + nifedipine, 50 mg/kg body weight (B.W)/day by gavage bid), SHR-CON (SHR + 0.9% saline), and SHR-NIF (SHR + nifedipine). All rats were housed in a temperature-controlled room (12 h light/dark cycle) with free access to water and standard rodent chow. Nifedipine (Yunpeng, Shanxi) was used at an effective antihypertensive dose.

### Non-invasive blood pressure measurements

BP and heart rate (HR) was measured in the warmed, restrained, conscious animals by using the tail-cuff method (BP-98A, Softron, Tokyo, Japan) as described previously^29^. All animals were acclimated to the apparatus for 3 days prior to recording, and measurements were taken at the same time. At least 5 successive measurements were recorded, and the average values were used to determine the resting arterial BP.

### Ultrasound imaging

Ultrasound imaging was performed by using a Vevo 2100 system (VisualSonics, Toronto, Canada) with a 16-MHz linear transducer (MS-250) before and after drug treatments. Rats were anesthetized with 2% isoflurane via nose cone at a flow rate of 1 L/min and placed in supine position on a heated platform to maintain a body temperature at 37 °C for continuous electrocardiogram (EKG) monitoring. To assess left ventricular (LV) function, M-mode images were performed in the short axis to calculate fractional shortening (FS), the ejection fraction (EF), stroke volume (SV) and cardiac output (CO). In an apical four chamber view, a pulsed wave (PW) Doppler was used to measure the early (E) and late (A) peak mitral flow velocity, and tissue Doppler imaging (TDI) was used to measure the early (E′) and late (A′) annular motion/velocity^30^. All Doppler spectra were recorded across at least 5 cardiac cycles at 200 mm/s sweep speed, and the parameters were assessed in 3 consecutive cardiac cycles. All data were collected and analysed offline by a single observer who was blinded to the treatment of the rats.

### PWV

To evaluate the PWV, PW Doppler images were obtained at the ascending aorta (just above the aortic valve, site 1) and the arch (just below the bifurcation of the left subclavian artery, site 2). The methodology was quantified as previously described^31^ and is available in the online-only Data Supplement.

### Vascular distension

Vascular distension was quantified as previously described^18^. Detailed methodology is available in the online-only Data Supplement.

### Invasive haemodynamic measurements

Invasive haemodynamic measurements was described as previously studies^32^. Detailed methodology is available in the online-only Data Supplement.

### Histology

The aorta was removed and fixed with 4% paraformaldehyde and dehydrated in 30% sucrose. In addition, the ascending aorta and aortic arch were separated and embedded in optimal cutting temperature medium in suitable EP tubes with markers that indicated the accurate position of the outer and inner arterial wall. After freezing the aorta at temperatures colder than −30 °C, it was sectioned at a thickness of 8 μm at intervals of 100 μm along the vessel. The middle sections of the ascending aorta and arch, in accordance with the locations of the ultrasonic testing in vascular distension, were collected. H&E staining as well as VVG elastin staining (GenMed Scientifics Inc., USA) were applied to detect the structure of the vessels. An image analysis was performed using Image-Pro Analyzer software (Media Cybernetics, USA) to quantify the aortic diameter, vessel wall thickness, elastin layer thickness and CSA.

### Geometry data acquisition of the rat aorta

Prior to euthanization, rats selected for scanning micro-CT (a SHRs and a WKYs) received vascular perfusions as previously described^33^. After anaesthesia, rats underwent a thoracotomy to allow access to the heart, and the descending aorta was exposed. A 24G butterfly needle attached to 0.75 in polyethylene tubing was inserted into the left ventricle, and the abdominal aorta was severed to provide a drainage point. The LV was flushed with 100 ml of heparinized normal saline (1000 U/ml) followed by 30 ml of 10% formalin fixation and then final perfusion with 10 ml of Microfil (MV-122; Flow Tech, Carver, Mass, USA) at a nominal pressure of 100 mmHg until the tongues and eyes were coloured by the Microfil. The Microfil compound polymerized at 4 °C for 24 hours. The thorax was carefully dissected and fixed with 10% neutral buffered formalin before micro-CT imaging.

Micro-CT imaging was performed using an Inveon MM CT (SIEMENS, Munich, Germany) system as described previously^34^. The specimens were located and scanned as a whole in each of the 360 rotational steps. Images were acquired at a voltage of 80 kV, a current of 500 μA, an effective pixel size of 9.08 μm and an exposure time of 1000 ms. The images consisted of 2048 × 3040 × 2048 voxels that equated to 9.08 μm × 9.08 μm × 9.08 μm per voxel, which produced 1535–1536 slices.

### Numerical models creation

The geometric data of the aorta were chosen for the SHRs and WKYs models based on the representative anatomical features and the high quality of the micro-CT imaging. Image segmentation and surface reconstruction of the aorta were accomplished by a semi-automatic threshold-based segmentation tool (Mimics17.0, Materialise Inc., Belgium). The models were exported into the stereolithographic (STL) file format after smoothing. The format of aorta models was changed into X_T by using the exact surface function (Geomagic Wrap2015, Geomagic Inc., USA). Detailed views of the two models are shown in Supplementary Fig. S5, and the size of the geometric model is shown in Supplementary Table S7.

### Meshing and elements

The reconstructed geometry was meshed by using HyperMesh v10.0 (Altair HyperWorks, Troy, MI, USA) with tetrahedral elements. The grid was divided into various entrances, exit and wall regions. The element numbers of the WKYs model and SHRs model were 739496 and 741028 (129161 and 130051 nodes), respectively.

### Boundary conditions and flow models

The Navier-Stokes equations were numerically solved with a commercial finite volume-based CFD solver (Fluent15.0, ANSYS, Inc., USA). Temporally adjusted velocity profiles (at site 1) were imposed at the inlet of the aorta based on the flow velocity waveforms obtained from the *in vivo* measurements^35^. On the basis of the lumen CSA ratio in geometric models, the flow volume ratios of the innominate, left common carotid, and left subclavian arteries were set as 10:7:8 (SHRs) and 9:6:10 (WKYs), respectively^36^. The curve of blood flow volume of the descending aorta was based on the velocity through the distal aortic arch (at site 2).

Transient analysis was adopted to investigate the pulsatile nature of blood flow. No-slip boundary conditions were assigned at the wall, and rigid wall models were used in all simulations. Blood was defined as incompressible, and blood has the same kinematic viscosity and density of a Newtonian fluid with a dynamic viscosity of 3.5 mPa·s and a density of 1060 kg/m^37^. The average Reynolds (Re) number at the aortic root is a dimensionless number reflecting the relative influence of transient inertial forces compared with that of viscous forces and was calculated as the following: Re = ρvd/μ, where ρ is the density of blood, v is the mean velocity, μ is the dynamic viscosity of blood, and d is the diameter of the vessel. The mean Reynolds numbers vary between 250 and 790, and the maximum Reynolds number is 1619 in our models. Then the blood flow is assumed to be laminar^38^. To ensure that initial transients would not be present in the solution, at least three cardiac cycles were simulated with 100 time steps included per cardiac cycle to ensure stability. The results presented here were obtained from the third cycle.

### Derived Haemodynamic Parameters

Derived haemodynamic parameters include the WSS, TAWSS, OSI and RRT. WSS is an analytical factor used to describe the dynamic friction between the viscous fluid and the solid wall, which is caused by the lateral movement of the viscous fluid. TAWSS is obtained by averaging the WSS in a cardiac cycle and is a better representative of WSS.

1 $$TAWSS=\frac{1}{T}{\int }_{0}^{T}WSSdt$$

We set 20 Pa as an interval and divided the values into 7 ranges to describe ﻿TAWSS. OSI reflects the cyclic departure of the WSS (or velocity) vector from predominant direction of blood flow^9^ and is calculated as equation (2):

2 $$OSI=\frac{1}{2}(1-\frac{|{\int }_{0}^{T}{\tau }_{\omega }dt|}{{\int }_{0}^{T}|{\tau }_{\omega }|dt})$$

where *τ*_*ω*_ is wall shear stress and T is one cardiac cycle. The OSI values vary from 0 to 0.5: 0 represents unidirectional flow, and 0.5 signifies complete oscillatory flow. RRT refers to the relative time for a particle stagnating in an area^9^.

3 $$RRT=\frac{1}{\delta }=1/\{(1-2OSI)TAWSS\}$$

### Statistics

The results are shown as the mean ± standard deviation (SD). ANOVA, non-parametric tests and linear regression analysis were used to analyse the data. The final measured values were loaded into SPSS 22.0 (SPSS Inc. Chicago, IL, USA) for statistical analysis. *P* values < 0.05 were considered as statistically significant.

## Electronic supplementary material

Supplementary methods and data
