# Supplementary material for: Vascular Remodelling Relates to an Elevated Oscillatory Shear Index and Relative Residence Time in Spontaneously Hypertensive Rats
Source: Sci Rep. 2017 May 17;7:2007. doi: 10.1038/s41598-017-01906-x (PMC5435712; doi:10.1038/s41598-017-01906-x)
Supplement: Supplementary file 1 — Supplementary methods and data [file 41598_2017_1906_MOESM1_ESM.pdf]

Vascular Remodelling Relates to an Elevated Oscillatory Shear Index and Relative Residence Time in Spontaneously Hypertensive Rats

Zhiyan Chen[1#], Haiyi Yu[1#], Yue Shi[2], Minjia Zhu[2], Yueshen Wang[1], Xi Hu[1], Youyi Zhang[1], Yu Chang[2]*, Ming Xu[1]*, Wei Gao[1]*

1 Department of Cardiology, Peking University Third Hospital and Key Laboratory of Cardiovascular Molecular Biology and Regulatory Peptides, Ministry of Health, Key Laboratory of Molecular Cardiovascular Sciences, Ministry of Education and Beijing Key Laboratory of Cardiovascular Receptors Research, Beijing 100191, China

2 College of Life Science and Bioengineering, Beijing University of Technology, Beijing 100124, China

# These authors contributed equally to this work.

Supplementary methods

Pulse wave velocity

Doppler imaging was performed at the ascending aorta (just above the aortic valve, site 1) and the aortic arch (just below the bifurcation of the left subclavian artery, site 2). The arrival time of the velocity wave is measured as the time from the peak of the EKG R-wave to the foot where the velocity begins to rise at the start of systole. Subsequently, the distance between these two points along the vessel was measured using a B-model. The PWV was calculated by dividing the measured distance by the time difference between the 2 arrival times at each location[1]. Each arrival time was assessed in 6-8 consecutive cardiac cycles, and distance was 3 consecutive cardiac cycles.

Vascular distension

An M-model was obtained on a parasternal long-axis view at two sites: the middle of the ascending aorta and the middle location between the two bifurcations in the aortic arch (the brachiocephalic artery and left common carotid artery). The diameters were measured during end-diastole (Dd) and during systole (Ds), respectively, using the inner edge method. In addition, vascular distension can be assessed using the following equation[2]: change rate = (Ds- Dd)/ Dd. Each diameter was assessed over 3 consecutive cardiac cycles.

Invasive haemodynamic measurements

Assessment of cardiac function was conducted by using a Scisense catheter (FTS-1611B-0018, Scisense, UK) as described previously[3]. After the rats were anaesthetized, the right carotid artery was exposed and ligated, and a 1.6 F catheter was inserted and advanced to the left ventricle. The maximum and minimum values of the first derivative of the LV pressure (+dP/dtmax and -dP/dtmax) were determined offline using a Biopac System (MP100, BIOPAC System, Inc., USA).

Reference

1. Tan, I., Butlin, M., Liu, Y. Y., Ng, K. & Avolio, A. P. Heart rate dependence of aortic pulse wave velocity at different arterial pressures in rats. *Hypertension*. **60**, 528-533 (2012).
2. Vayssettes-Courchay, C., Ragonnet, C., Isabelle, M. & Verbeuren, T. J. Aortic stiffness in vivo in hypertensive rat via echo-tracking: analysis of the pulsatile distension waveform. *Am J Physiol Heart Circ Physiol*. **301**, H382-390 (2011).
3. Sun, Y. *et al.* Deletion of inducible nitric oxide synthase provides cardioprotection in mice with 2-kidney, 1-clip hypertension. *Hypertension*. **53**, 49-56 (2009).

Supplementary data

Supplementary Table S1 Baseline of the blood pressure in SHRs and WKYs.

	CON		NIF	
	WKY-CON	SHR-CON	WKY-NIF	SHR-NIF
W (g)	415 ± 14	366 ± 23***	413 ± 14	375 ± 12***††
HR (beats/min)	302 ± 34	369 ± 20**	302 ± 24	359 ± 26*†
SBP (mmHg)	128 ± 2	218 ± 17***	131 ± 10	209 ± 5***†††
DBP (mmHg)	88 ± 9	171 ± 17***	95 ± 10	159 ± 10***†††
PP (mmHg)	40 ± 8	51 ± 8 **	36 ± 4	50 ± 7*††

Values are presented as the mean ± SD. (n = 6). **P* < 0.05, ***P* < 0.01, ****P* < 0.001, *vs* WKY-CON. †*P* < 0.05, ††*P* < 0.01, †††*P* < 0.001, *vs* WKY-NIF. W, weight; HR, heart rates; SBP, systolic blood pressure; DBP, diastolic blood pressure; PP, pulse pressure. CON, control; NIF, nifedipine.

Supplementary Table S2 Characteristics of cardiac function in SHRs and WKYs.

	CON		NIF	
	WKY-OCN	SHR-CON	WKY-NIF	SHR-NIF
LVAW (mm)	1.87 ± 0.2	2.12 ± 0.37	1.89 ± 0.28	2.35 ± 0.42*†
LVPW (mm)	1.90 ± 0.09	2.19 ± 0.12**	1.86 ± 0.22	2.14 ± 0.18*††
LVESV (μl)	91 ± 43	117 ± 36	81 ± 40	77 ± 39
LVEDV (μl)	309 ± 67	341 ± 78	323 ± 62	303 ± 66
SV (μl)	218 ± 30	223 ± 51	242 ± 28	226 ± 31
CO (ml/min)	67 ± 10	70 ± 20	67 ± 12	72 ± 8
EF (%)	72 ± 9	66 ± 6	76 ± 8	76 ± 8#
FS (%)	43 ± 7	38 ± 5	47 ± 7	47 ± 7#
MV E/A	1.67 ± 0.32	1.7 ± 0.6	1.85 ± 0.36	1.54 ± 0.2
MV E/E'	25 ± 4	31 ± 10	26 ± 1	33 ± 6**†
+dp/dtmax	7669 ± 1066	8570 ± 2255	9036 ± 3005	9543 ± 910
-dp/dtmax	8389 ± 4234	7057 ± 1259	9423 ± 2535	11842 ± 3370#

Values are presented as the mean ± SD. (n = 6). *P < 0.05, **P < 0.01, *vs* WKY-CON. #P < 0.05, *vs* SHR-CON. †P < 0.05, ††P < 0.01, *vs* WKY-NIF. LVAW, left ventricular anterior wall; LVPW, left ventricular posterior wall; LVESV, left ventricular end-systolic volume; LVEDV, left ventricular end-diastolic volume; EF, ejection fraction; FS, fractional shortening; MV E/A, mitral valve E/A; MV E/E', mitral valve E/E'; +dp/dtmax, maximal rate of pressure rise; -dp/dtmax, maximal rate of pressure fall; SV, stroke volume; CO, cardiac output; CON, control; NIF, nifedipine.

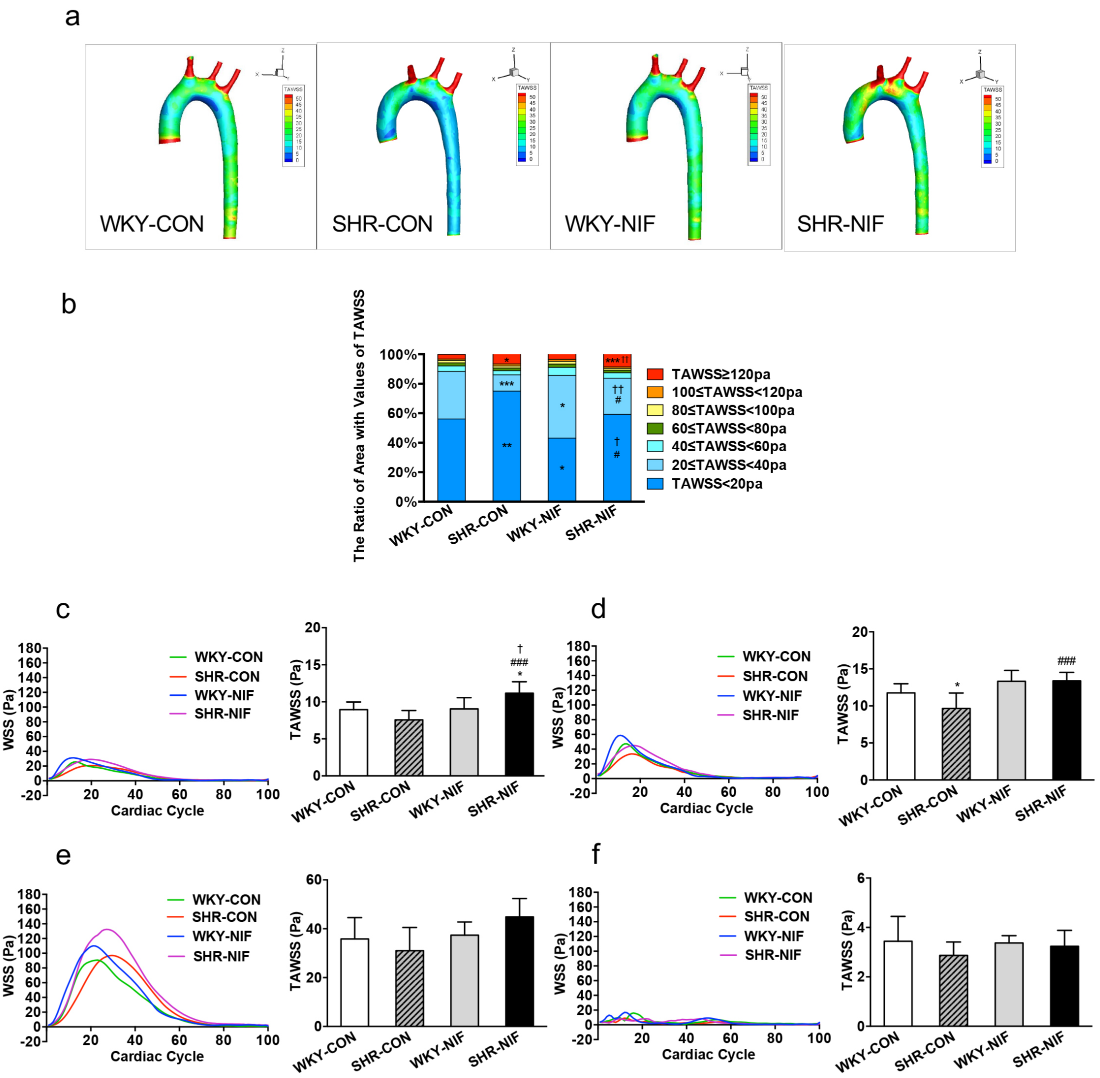

Supplementary Fig. S1 TAWSS was improved in antihypertensive-treated SHRs. (a) TAWSS contours averaged over a cardiac cycle computed from CFD models of the aortas in SHRs and WKYs. The TAWSS scale ranges from 0 (dark blue) to 50 Pa (Red). (b) For the whole models, the analysis based on the ratio of the area with different TAWSS values were shown. In specific regions, the instantaneous WSS variance during one cardiac cycle and the TAWSS values were represented near the (c) outer wall and (d) inner wall of the ascending aorta and the (e) outer wall and (f) inner wall of the aortic arch. Values are presented as the mean ± SD. (n = 6). $^{*}P < 0.05$, $^{**}P < 0.01$, $^{***}P < 0.001$, *vs* WKY-CON. $^{\#}P < 0.05$, $^{\#\#\#}P < 0.001$, *vs* SHR-CON. $^{\dagger}P < 0.05$, $^{\dagger\dagger}P < 0.01$, *vs* WKY-NIF. CON, control; NIF, nifedipine.

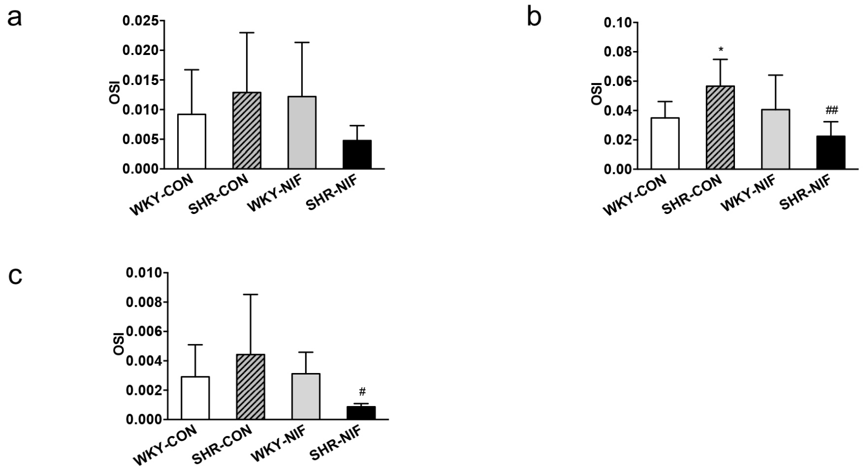

Supplementary Fig. S2 The OSI values were decreased in the regions in antihypertensive-treated rats. The OSI values are shown near (a) the outer and (b) the inner wall of the ascending aorta and (c) the outer wall of the aortic arch. Values are presented as the mean ± SD. (n = 6). **P* < 0.05, *vs* WKY-CON. #*P* < 0.05, ##*P* < 0.01, *vs* SHR-CON. CON, control; NIF, nifedipine.

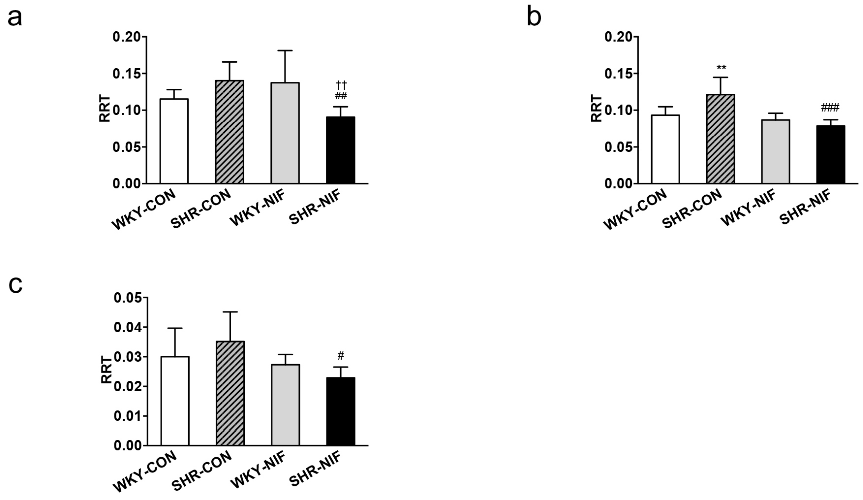

Supplementary Fig. S3 The RRT values were decreased in the regions in antihypertensive-treated rats. The RRT values are shown near (a) the outer and (b) the inner wall of the ascending aorta and (c) the outer wall of the aortic arch. Values are presented as the mean ± SD. (n = 6). $^{**}P < 0.01$, *vs* WKY-CON. $^{\#}P < 0.05$, $^{\#\#}P < 0.01$, $^{\#\#\#}P < 0.001$, *vs* SHR-CON. $^{\dagger\dagger}P < 0.01$, *vs* WKY-NIF.

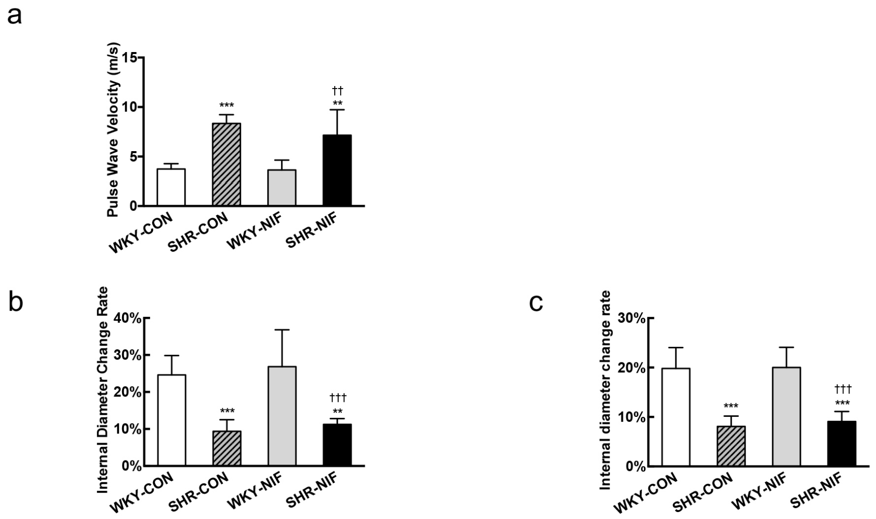

Supplementary Fig. S4 The aortic function was significant impaired in hypertensive and antihypertensive-treated SHRs. (a) The PWV was significantly increased, and the distension (ΔD/D) of (b) the ascending aorta and (c) the aortic arch were decreased in SHRs. Values are presented as the mean ± SD. (n = 6). $^{**}P < 0.01$, $^{***}P < 0.001$, *vs* WKY-CON. $^{††}P < 0.01$, $^{†††}P < 0.001$, *vs* WKY-NIF. CON, control; NIF, nifedipine.

Supplementary Table 3 Characteristics of the aorta determined at the level of the ascending aorta and aortic arch tissue sections in SHRs and WKYs.

		CON		NIF	
		WKY-CON	SHR-CON	WKY-NIF	SHR-NIF
AS	CSA (mm^2)	0.99 ± 0.16	1.56 ± 0.10***	1.09 ± 1.09	1.63 ± 0.18***†††
	D (mm)	1.75 ± 0.17	2.20 ± 0.16***	1.92 ± 0.07	2.23 ± 0.19***††
	TLR	0.38 ± 0.08	0.38 ± 0.05	0.35 ± 0.35	0.38 ± 0.06
AO	CSA (mm^2)	1.38 ± 0.22	2.12 ± 0.24***	1.36 ± 1.36	2.23 ± 0.34***†††
	D (mm)	1.94 ± 0.13	2.32 ± 0.08**	2.00 ± 0.11	2.41 ± 0.17**††
	TLR	0.42 ± 0.04	0.45 ± 0.04	0.39 ± 0.39	0.44 ± 0.04

Values are presented as the mean ± SD. (n = 6). ***P* < 0.01, ****P* < 0.001, *vs* WKY-CON. ††*P* < 0.01, †††*P* < 0.001, *vs* WKY-NIF. AS, ascending aorta; AO, aortic arch; CSA, cross-sectional area; D, luminal diameter; TLR, thickness lumen rate. CON, control; NIF, nifedipine.

Supplementary Table 4 Analysis of the elastin layer at the level of ascending aorta and arch tissue sections in SHRs and WKYs.

		CON		NIF	
		WKY-CON	SHR-CON	WKY-NIF	SHR-NIF
AS	CSA_E (mm^2)	0.76 ± 0.08	1.17 ± 0.10***	0.78 ± 0.07	1.22 ± 0.23***†††
	T_{IW} (mm)	0.15 ± 0.01	0.23 ± 0.02***	0.15 ± 0.01	0.23 ± 0.04***†††
	T_{OW} (mm)	0.14 ± 0.02	0.15 ± 0.02	0.13 ± 0.02	0.16 ± 0.03†
AO	CSA_E (mm^2)	0.88 ± 0.11	1.58 ± 0.14***	0.86 ± 0.05	1.69 ± 0.33***†††
	T_{IW} (mm)	0.19 ± 0.02	0.25 ± 0.04*	0.17 ± 0.01	0.27 ± 0.04*†††
	T_{OW} (mm)	0.10 ± 0.01	0.15 ± 0.02**	0.10 ± 0.01	0.15 ± 0.03***††

Values are presented as the mean ± SD. (n = 6). **P* < 0.05, ***P* < 0.01, ****P* < 0.001, *vs* WKY-CON. †*P* < 0.05, ††*P* < 0.01, †††*P* < 0.001, *vs* WKY-NIF. AS, ascending aorta; AO, aortic arch; CSA_E, cross-sectional area of extensive elastin degradation; T_{IW}, thickness of elastin layer near the inner wall; T_{OW}, thickness of elastin layer near the outer wall; CON, control; NIF, nifedipine.

Supplementary Table 5 The correlations between haemodynamic parameters and vascular remodelling parameter (vessel wall thickness) near the outer and the inner wall of ascending aorta and the outer wall of aortic arch in SHRs and WKYs.

	OSI		RRT	
	r	*P*	r	*P*
**OWAS**	-0.007	0.975	-0.091	0.674
**IWAS**	-0.054	0.803	0.114	0.597
**OWAO**	-0.068	0.752	0.127	0.555

r, linear regression coefficient between haemodynamic parameters and vascular remodelling parameter. OWAS, the outer wall of ascending aorta; IWAS, the inner wall of ascending aorta; OWAO, the outer wall of aortic arch.

Supplementary Table 6 The correlations between haemodynamic parameters and vascular remodelling parameter (elastin layer thickness) near the outer and the inner wall of ascending aorta and the outer wall of aortic arch in SHRs and WKYs.

	**OSI**		**RRT**	
	**r**	***P***	**r**	***P***
**OWAS**	-0.277	0.190	0.089	0.681
**IWAS**	-0.244	0.251	0.090	0.675
**OWAO**	-0.159	0.458	-0.044	0.839

r, linear regression coefficient between haemodynamic parameters and vascular remodelling parameter. OWAS, outer wall of ascending aorta; IWAS, inner wall of ascending aorta; OWAO, outer wall of aortic arch.

Supplementary Table 7 Geometric model size of the branches of blood vessels.

Locations	Area (mm^2)	
	WKYs	SHRs
Ascending aorta (①)	3.77	4.64
Brachiocephalic artery (②)	0.32	0.38
Left common carotid artery (③)	0.24	0.26
Left subclavian artery (④)	0.38	0.32
The distal aortic arch (⑤)	3.98	4.26
Descending aorta (⑥)	1.54	1.81

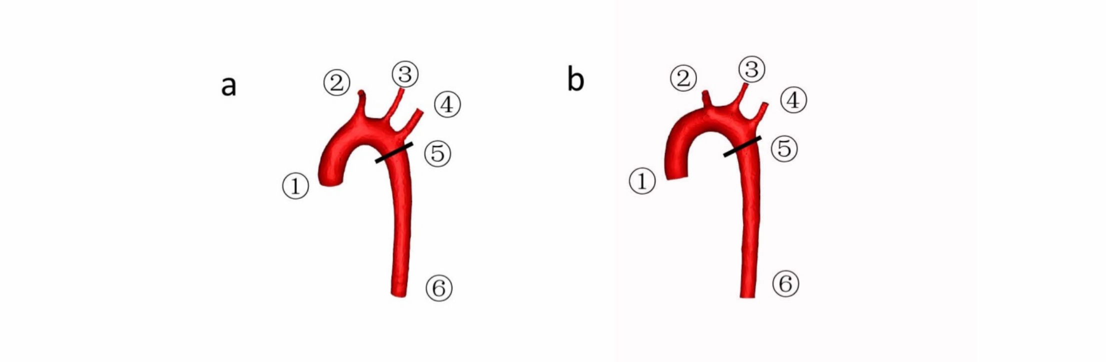

Supplementary Fig. S5 Digital models in (a) WKYs and (b) SHRs.
